# Targeted Interventions to Increase Blood Pressure and Decrease Anaesthetic Concentrations Reduce Intraoperative Burst Suppression: A Randomised, Interventional Clinical Trial

**DOI:** 10.3389/fnsys.2022.786816

**Published:** 2022-03-04

**Authors:** Marie-Therese Georgii, Matthias Kreuzer, Antonia Fleischmann, Jule Schuessler, Gerhard Schneider, Stefanie Pilge

**Affiliations:** Department of Anaesthesia and Intensive Care, Klinikum rechts der Isar, Technical University of Munich, Munich, Germany

**Keywords:** Burst Suppression Rate, entropy, intraoperative neuromonitoring, anaesthetic intervention, electroencephalography

## Abstract

**Background:**

It has been suggested that intraoperative electroencephalographic (EEG) burst suppression (BSupp) may be associated with post-operative neurocognitive disorders in the elderly, and EEG-guided anaesthesia may help to reduce BSupp. Despite of this suggestion, a standard treatment does not exist, as we have yet to fully understand the phenomenon and its underlying pathomechanism. This study was designed to address two underlying phenomena—cerebral hypoperfusion and individual anaesthetic overdose.

**Objectives:**

We aimed to demonstrate that targeted anaesthetic interventions—treating intraoperative hypotension and/or reducing the anaesthetic concentration—reduce BSupp.

**Methods:**

We randomly assigned patients to receive EEG-based interventions during anaesthesia or EEG-blinded standard anaesthesia. If BSupp was detected, defined as burst suppression ratio (BSR) > 0, the primary intervention aimed to adjust the mean arterial blood pressure to patient baseline (MAP intervention) followed by reduction of anaesthetic concentration (MAC intervention).

**Results:**

EEG-based intervention significantly reduced total cumulative BSR, BSR duration, and maximum BSR. MAP intervention caused a significant MAP increase at the end of a BSR > 0 episode compared to the control group. Coincidentally, the maximum BSR decreased significantly; in 55% of all MAP interventions, the BSR decreased to 0% without any further action. In the remaining events, additional MAC intervention was required.

**Conclusion:**

Our results show that targeted interventions (MAC/MAP) reduce total cumulative amount, duration, and maximum BSR > 0 in the elderly undergoing general anaesthesia. Haemodynamic intervention already interrupted or reduced BSupp, strengthening the current reflections that hypotension-induced cerebral hypoperfusion may be seen as potential pathomechanism of intraoperative BSupp.

**Clinical Trial Registration:**

NCT03775356 [ClinicalTrials.gov], DRKS00015839 [German Clinical Trials Register (Deutsches Register klinischer Studien, DRKS)].

## Introduction

Intraoperative electroencephalographic (EEG) burst suppression (BSupp) is a non-specific and non-physiological EEG pattern.

The occurrence of BSupp has often been attributed to a relative “overdose” of volatile or intravenous anaesthetics (Bruhn et al., 2000b). This may not necessarily be related to high absolute concentrations, and the occurrence at lower concentrations suggests a vulnerability or increased cerebral sensitivity to (predominantly volatile) anaesthetics in patients at risk (Fritz et al., 2018). Additionally, pathophysiological aspects such as hypotension-induced cerebral hypoperfusion may also been considered as potential cause of BSupp (Sessler et al., 2012).

Several studies suggested an association between BSupp and post-operative neuro-cognitive disorders (pNCD). More specifically, BSupp is considered as a possible predictor of post-operative delirium (POD) (Radtke et al., 2013; Soehle et al., 2015; Fritz et al., 2016). pNCD is a common post-operative complication, particularly in the elderly, and is associated with increased morbidity and mortality. The evidence-based and consensus-based guidelines on POD of the European Society of Anaesthesiology (ESA) recommend (grade A) EEG-based anaesthesia monitoring for all patients to avoid too deep general anaesthesia, i.e., BSupp (Aldecoa et al., 2017).

In a clinical context, the information of an “excessively deep hypnotic level” is often presented by a processed EEG (pEEG) index called (Burst) Suppression Ratio [(B)SR]. This index represents the percentage of suppressed EEG within a defined time span (Rampil, 1998; Bruhn et al., 2000a).

Previous studies described a correlation between BSupp and POD or a reduction of POD when EEG-monitoring was applied (Radtke et al., 2013; Whitlock et al., 2014; Fritz et al., 2016, 2018; MacKenzie et al., 2018). But none of these studies had the primary goal to study the influence of BSupp reduction on POD or pNCD. Hence, the observed correlation may reflect an epiphenomenon. To test the primary hypothesis whether BSupp was related to POD, Wildes et al. (2019) recently conducted a randomised interventional trial (ENGAGES). Their findings, however, could not prove that EEG-guided anaesthetic interventions such as reducing anaesthetic administration and minimising EEG suppression decreases the incidence of POD when compared to routine anaesthesia care (Wildes et al., 2019). Nevertheless, it showed that reduced anaesthetic concentration resulted in shorter time spent in BSupp.

In summary, the impact of BSupp on pNCD is still unclear and controversially discussed.

We set out to design an interventional approach to reduce BSupp by reducing the total cumulative BSR through a targeted regimen to systematically investigate the influence of MAP and MAC interventions on BSupp and (secondary) pNCD. These results may help to design intervention-based protocols that focus on a possible causality between BSupp and pNCD.

The interventions were applied stepwise and consisted of (1) treatment of intraoperative hypotension and (2) reduction of anaesthetic concentration, if BSR occurred.

## Materials and Methods

### Design, Ethics, and Outcomes

We conducted a single centre, (single)-blinded, randomised, interventional clinical trial approved by the local ethics committee (Chairperson Georg Schmidt; approved 13 August, 2018). All patients received detailed information and provided their written informed consent. The trial was conducted from January 2019 until December 2020 and included 110 screened patients. The primary outcome was a reduction of the total cumulative BSR. As a secondary outcome, we investigated whether a mean arterial blood pressure (MAP) and/or minimal alveolar concentration (MAC) intervention resulted in a reduced BSR. Further, we collected data regarding POD incidence.

### Patient Population - Patient Database and Criteria

We focused on patients aged ≥60 years as they seem more susceptible to BSupp and are at higher risk of developing POD (Radtke et al., 2013; Fritz et al., 2016). The inclusion criteria contained all interventions under general anaesthesia (volatile/balanced and total-intravenous) of at least 60 min expected surgical duration and patients of all ASA (American Society of Anaesthesiology) scores. We excluded patients who met the following criteria: unable to provide informed consent, hearing impaired, not fully orientated, not fluent in German, pre-existing neuro-psychiatric diseases, cranial or otolaryngeal surgery and planned post-operative admission to ICU, or prolonged respiratory assistance.

### Randomisation and Blinding

Patients were randomised in blocks of 22 in 5 sections, using paper-envelopes containing notes assigning them to either EEG-blinded, standard anaesthesia care (control group, CNT) or to EEG-guided anaesthesia (intervention group, INT). Randomisation was performed on the day of surgery prior to entering the induction room. We did not present the (processed) EEG information to the anaesthesiologist for the control group. In the intervention group both the EEG-based indices and the EEG were displayed perioperatively.

### Procedures/Interventions

**FIGURE 1:**
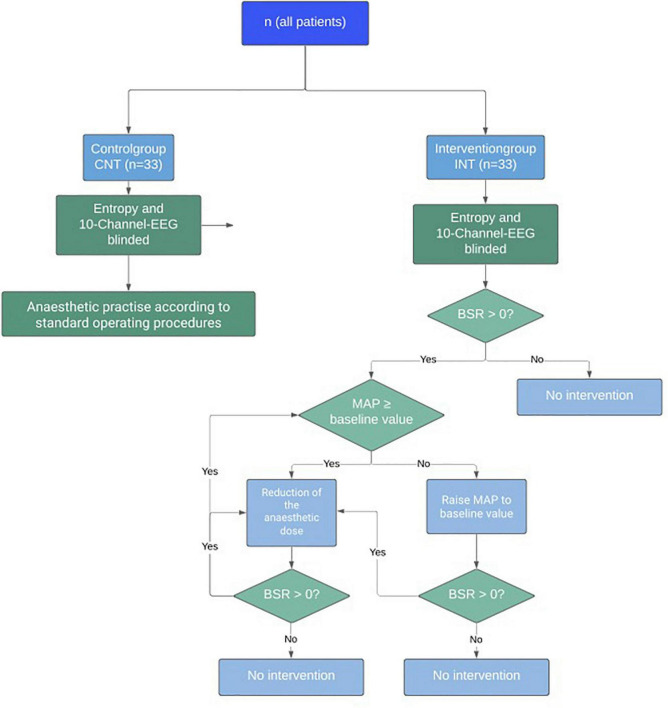
Study algorithm for the interventional trial on reduction of intraoperative Burst Suppression Rate (BSR).

While the control group was anaesthetised according to standard operating procedures (SOPs), the intervention group was treated according to a given algorithm when BSR > 0. First, when BSR > 0, we compared the MAP to the baseline MAP(BL), defined as the lowest MAP observed in the time before surgery, i.e., during pre-anaesthesia visit, on the ward, in the induction room, and before starting the induction. If BSupp occurred while MAP was below baseline value, the blood pressure level was increased primarily by catecholamines (Norepinephrine, Theodrenalin-Cafedrin) according to the hospital’s SOPs (MAP intervention). Second, when BSR remained positive after MAP intervention, we reduced the anaesthetic concentration until BSR = 0% (MAC intervention). Figure 1 visualizes the algorithm.

The anaesthesiologist managed the anaesthetic procedure independently according to SOPs and was only instructed by the trial team in case of BSR > 0.

### Data Collection

We recorded the pEEG parameters response and state entropy (RE/SE), and the BSR with the GE Entropy Module (GE Healthcare, Helsinki, Finland). We additionally recorded 10-channel EEG, using the Medtronic NIM Eclipse System for further analysis. We focused on reducing the BSR by targeted interventions and defined *BSR* > 0 as the primary trigger to intervene. From our electronic records that were stored as .csv files, we extracted the BSR and SE, the (invasively measured) MAP, the endtidal MAC values with 10 s resolution, all given doses of intravenous anaesthetics, and the time points of recorded events. In case of a non-invasive blood pressure (BP) measurement the trend data resolution was 2–3 min.

We examined each patient for delirium using the modified brief cognitive assessment method (bCAM) (Ely et al., 2001). We screened all patients in the post anaesthesia care unit (PACU) 15 and 45 min after emergence and once a day during the first three post-operative days. We chose the bCAM in light of the limited amount of time and the examiner’s background, as it reliably detects both hypo- and hyper-active delirium and showed best evidence of use (Wong et al., 2010; van Velthuijsen et al., 2016).

A defined nomenclature of cognitive changes associated with anaesthesia and surgery is still not conclusively existent. Hence, in this trial one positive bCAM of two consecutive assessments in the PACU was defined as “PACU-delirium” due to its early post-operative onset and temporary duration. In cases of ongoing or delirious symptoms during the first three post-operative days, we diagnosed delirious patients with “POD.”

### Burst Suppression Ratio Algorithm

While SE and RE reflect the hypnotic component of anaesthesia (Viertio-Oja et al., 2004), BSR is based on the detection of suppressed EEG episodes by using a special algorithm (Särkelä et al., 2002).

From the extracted BSR trend data we identified occurrences of BSupp as BSR > 0 and calculated the following parameters: (i) BSR duration, (ii) mean BSR, (iii) maximum BSR, and (iv) the cumulative BSR. The total cumulative BSR was defined by the area under the BSR trend curve. Hence, it includes duration and intensity.

### Analysis of Burst Suppression Ratio, Mean Arterial Blood Pressure, Minimal Alveolar Concentration, and Anaesthetic Doses

For the primary outcome, we compared BSR variables between the two groups considering the entire anaesthetic procedure.

For the secondary outcome, we focused on the occurrence of BSR > 0 during induction or maintenance. The induction phase began with administering the first anaesthetic and ended with clearance. We defined the maintenance period as the time from clearance for surgery until the end of surgery. In two cases, the end of surgery was not documented, and we used the termination of the recording as endpoint.

We evaluated the number of MAP and MAC interventions and their effect on BSR. We determined MAP and endexpiratory MAC at the beginning of a positive BSR episode (MAPstart/MACstart) and when BSR returned to 0 (MAPend/MACend). Additionally, we compared these MAP values to the baseline MAP within each group. For induction we extracted the total amount of intravenous doses of propofol and sufentanil regarding single and repetitive applications.

We defined statistical exclusion criteria to compare the parameters between the two groups. These criteria were chosen arbitrarily according to the best of our clinical knowledge. Assuming that an intervention reveals a reasonable effect within 2 min, we only included BSR episodes of at least a 2-min duration. Since BSR calculation is based on at least 60 s of EEG, we defined two episodes interrupted by less than 60 s as one episode.

### Statistical Analysis

#### Power Analysis/Sample Size Calculation

To estimate the approximate incidence of a positive BSR in patients ≥60 years, we retrospectively analysed 8888 anaesthetic procedures recorded from January to August 2018. 4416/8888 patients showed a BSR > 0. Based on this pre-analysis, we assumed a 70% likelihood that the total cumulative BSR in the intervention group will be less than in the control group. Based on this assumption, a two-sided Mann-Whitney-*U*-Test obtains 80% power to detect this effect size of 70% at 0.05 significance level with a sample size of 2 × 33=66 patients. We analysed all outcomes using the intention-to-treat paradigm (Noether, 1987).

#### Descriptive and Inferential Statistics

We used the Mann-Whitney-*U*-Test to test for differences in parameter values and demographic characteristics between the control and the intervention group. Because of non-parametric testing, we did not test for normality. We present our data as box and scatter plots. We also calculated the area under the receiver operating curve (AUC) with 10k-fold bootstrapped 95%-confidence intervals using the MES toolbox (Hentschke and Stüttgen, 2011). For “within group” comparisons of MAPstart/MACstart and MAPend/MACend, we used the Wilcoxon signed rank test. In order to support the results of these tests with an effect size as well, we calculated the Hedge’s *g* value together with 10k-fold bootstrapped 95% confidence intervals also using the MES toolbox. We also calculated the difference between medians and constructed 95% confidence intervals with 1000-fold bootstrapping. In case 0 is not included within this confidence interval, the median difference between the two groups is significantly different. We used MATLAB (R2017a, The Mathworks, Natick, MA, United States) for statistical testing and graphical representation. For the scatter plots we used the *plotSpread* function from mathworks.com. To compare observed frequencies, we used the χ2 or the Fisher exact test from Social Science Statistics^1^.

## Results

**FIGURE 2:**
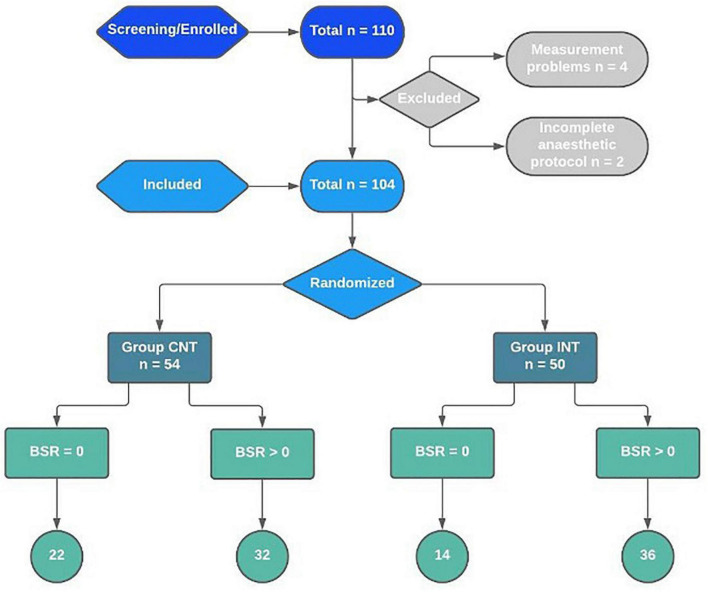
Recruitment, randomisation, and patient flowchart for the interventional trial on reduction of intraoperative BSR.

Of the 110 patients screened, we excluded four cases due to technical problems of the EEG setup. Hence, a total of 106 patients were enrolled. Due to two incomplete anaesthetic protocols, 104 data sets remained (CNT: 54 patients, INT: 50 patients). In accordance with our primary objective, we assessed 68 patients with a positive BSR [CNT: 32 patients (originally 33 minus 1 of the missing protocols), INT: 36 patients] (Figure 2).

The median age, BMI, and the duration of anaesthesia did not significantly differ between these groups (Supplementary Table 1). The majority of the patients were classified ASA2 (CNT: 23%, INT:26%) and ASA3 (CNT: 20%, INT:24%) without a significant difference between the groups (Supplementary Table 1). The median baseline MAP in the control group was not significantly different [CNT: 113 (103; 130) mmHg; INT: 109 (100; 116) mmHg; *p* = 0.156].

Patients predominantly underwent orthopaedic, urologic, or visceral surgery. Supplementary Table 2 shows the distribution of surgical disciplines amongst the patients.

### Primary Outcome

#### Reduction of Total, Cumulative Burst Suppression Ratio

**FIGURE 3:**
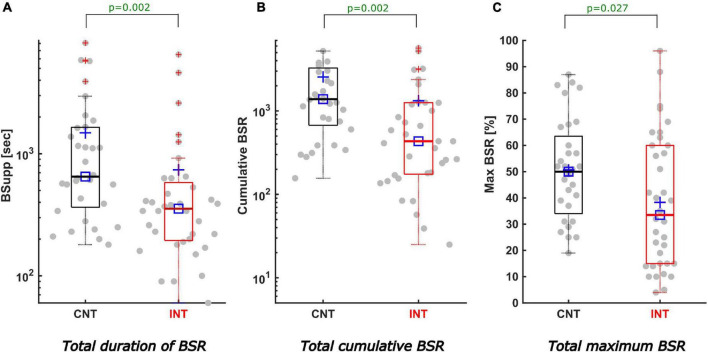
Description of total cumulative BSR, duration, and maximum of BSR. All boxplots show the medians (solid horizontal lines) and interquartile ranges (IQR, boundaries of the boxes). The square [□] indicates the median and the cross [+] the mean value. The dots represent all measured cases with positive BSR (68 patients) including outliers at the most extreme values outside the boxes. The values on the y-axis are calculated as logarithms. Black coloured boxes/graphs represent the control group and red coloured the intervention group. **(A)** Displays the total duration of BSR in seconds. The duration of positive BSupp was significantly less in the INTgroup (5.9 min) compared to the CNTgroup (10.1 min) (*p* = 0.002; difference of medians CI: 165–840 s). **(B)** The BSR(tot)–the sum of all positive BSR-values–was significantly reduced by intervention (CNTmedian 1385, INTmedian 433; *p* = 0.002; difference of medians CI: 281–1876). **(C)** Features a significant reduction of the maximum BSR-value (in %) in the INTgroup (CNTmedian 50, INTmedian 35; *p* = 0.027; difference of medians CI: -3–29.5).

The total, cumulative BSR was significantly lower in the intervention group [CNT: 1385 (673; 3270); INT:433 (175; 1256), *p* = 0.002; difference of medians CI: 281–1876]. Correspondingly, the median BSR duration was significantly reduced in the intervention group [CNT: 10.1 (6.1; 27.4) min; INT: 5.9 (3.3; 9.6) min, *p* = 0.002; difference of medians CI: 165–840 s], as was the maximum BSR [CNT: 50 (34; 64); INT: 35 (15; 60), *p* = 0.027; difference of medians CI: –3–29.5]. Figure 3 presents the boxplots for these analyses. Table 1 contains the details.

**TABLE 1:** Unpaired analysis of all characteristics (BSR, anaesthetic doses, MAP) between the groups while BSR > 0.

All Patients with positive BSR *n* = 68 Unpaired statistical analysis					
Perioperative measure	CNT (*n* = 32)	INT (*n* = 36)	*P*-Value	AUC	95% CI
**Patients with positive BSR (total quantity)**					
Only during induction	18	20			
Only during maintenance	5	11			
During induction and maintenance	9	5			
**Measures of total BSR**					
Total max BSR	50 [34; 64]	35 [15; 60]	0.027	0.66	[0.52; 0.78]
Total, cumulative BSR	1385 [673; 3270]	433 [175; 1256]	0.002	0.72	[0.60; 0.84]
Duration of BSR (min)	10.1 [6.1; 27.4]	5.9 [3.3; 9.6]	0.002	0.72	[0.59; 0.83]
**Anaesthetic duration (Int.–Ext.; min)**	173 [138; 209]	160 [127; 205]	0.358		
**Characteristics during INDUCTION**					
***BSR***					
Max BSR	42 [30; 64]	35 [15; 60]	0.177	0.61	[0.45;0.76]
Cumulative BSR	1184 [363; 2367]	373 [136; 1197]	0.011	0.70	[0.56; 0.83]
Duration of BSR (min)	5.8 [3.3; 12.3]	3.5 [2.2; 6.3]	0.029	0.68	[0.53; 0.81]
***MAP***					
MAP at start of BSR	84 [77; 96]	89 [83; 103]	0.166	0.39	[0.24; 0.55]
MAP at end of BSR	83 [75; 99]	105 [99; 117]	<0.001	0.19	[0.08; 0.32]
ΔMAP (end-start)	–2.9 [–16; 18]	13 [–7; 29]	0.033	0.33	[0.19; 0.48]
MAP ratio “start/end”	0.97 [0.84; 1.21]	1.17 [0.94; 1.35]	0.033	0.33	[0.19; 0.48]
MAP ratio “start/BL”	0.77 [0.64; 0.82]	0.84 [0.74; 0.96]	<0.001	0.30	[0.17; 0.45]
MAP ratio “end/BL”	0.77 [0.64; 0.83]	0.98[0.87; 1.10]	<0.001	0.15	[0.06; 0.28]
***Anaesthetic doses***					
**Induction dose of anaesthetics**					
Propofol “total” bolus(es) (mg)	200 [173; 245]	200 [150; 250]	0.361		
N with single bolus	7	11	0.254		
N with repetitive boli	20	15			
Sufentanil bolus(es) (mcg)	20 [15; 20]	20 [15; 20]	0.670		
**Characteristics during MAINTENANCE**					
***BSR***					
Max BSR	34 [13; 57]	27 [17; 43]	0.926	0.52	[0.25; 0.78]
Cumulative BSR	2129 [387; 2893]	1259 [403; 2892]	0.828	0.53	[0.29; 0.78]
Duration of BSR (min)	13.3 [4.8; 32.7]	10.8 [6.7; 21.3]	0.687	0.55	[0.30; 0.82]
Relative BSR	0.08 [0.04; 0.28]	0.09 [0.05; 0.19]	0.975	0.49	[0.25; 0.74]
***MAP***					
MAP at start of BSR	83 [78; 103]	82 [75; 91]	0.515	0.59	[0.33, 0.82]
MAP at end of BSR	82 [80; 93]	93 [85; 106]	0.114	0.30	[0.09; 0.54]
ΔMAP (start-end)	–1 [–3.2; 0.1]	0.7 [0.0; 5.2]	0.006	0.19	[0.04; 0.38]
MAP ratio “start/end”	0.96 [0.86; 1.02]	1.08 [1.00; 1.20]	0.012	0.19	[0.02; 0.39]
MAP ratio “start/BL”	0.76 [0.61; 0.83]	0.79 [0.66; 0.89]	0.789	0.46	[0.21; 0.73]
MAP ratio “end/BL”	0.71 [0.67; 0.75)	0.89 [0.75; 0.96]	<0.001	0.21	[0.04; 0.43]
***Anaesthetic doses–MAC***					
MAC at start of BSR	0.90 [0.81; 1.01]	1.05 [1.00; 1.25]	0.068	0.24	[0.03; 0.49]
MAC at end of BSR	0.96 [0.80; 1.03]	0.70 [0.60; 0.93]	0.052	0.77	[0.51; 1.00]
MAC ratio “start/end”	0.97 [0.93; 1.12]	0.60 [0.59; 1.06]	0.129	0.81	[0.54; 1.00]

### Secondary Outcome

#### Reduction of Burst Suppression Ratio During Induction

**FIGURE 4:**
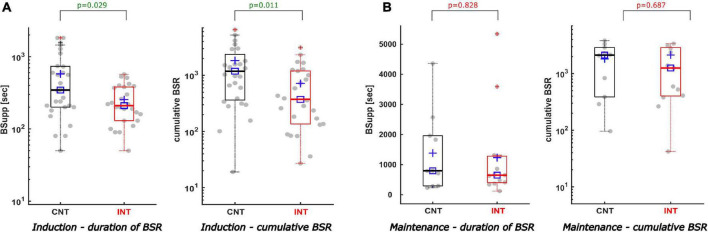
Description of the duration of BSR and the cumulative BSR separating induction from maintenance. **(A)** During induction the duration of BSR (A-left) and the cumulative BSR (A-right) were reduced significantly in the intervention group compared to the control group. The duration of BSR was cut by 2.3 min [*p* = 0.029, AUC 0.68; CI (0.53; 0.81)]. Similar reductive effect was achieved by intervention regarding the cumulative BSR during induction [CNTmedian 1184 vs. INTmedian 373; *p* = 0.011; AUC 0.70; CI (0.56; 0.83)]. **(B)** During maintenance the intervention did not produce statistically significant effects, nevertheless both the duration of BSR (B-left) and the cumulative BSR (B-right) demonstrate a decreasing trend in the intervention group [CNTmedian duration 13.3 min vs. INTmedian duration 10.8 min; *p* = 0.687; AUC 0.55; CI (0.30; 0.82)] [CNTmedian cumBSR 2129 vs. INTmedian cumBSR 1259; *p* = 0.828; AUC 0.53; CI (0.29; 0.78)].

During induction, the interventions led to a significant decrease in the cumulative BSR [CNT: 1184 (363; 2367); INT: 373 (136; 1197), *p* = 0.011] and the BSR duration [CNT: 5.8 (3.3; 12.3) min; INT 3.5 (2.2; 6.3) min, *p* = 0.029]. Figure 4A contains the corresponding boxplots. The maximum BSR value was 42 (30; 64) in the control group and 35 (15; 60) in the intervention group (*p* = 0.177). For induction, both groups received the same median amount of propofol [CNT: 200 (173; 245) mg; INT 200 (150; 250) mg; *p* = 0.361] and sufentanil [CNT: 20 (15; 20) μg; INT 20 (15; 20) μg; *p* = 0.670]. Further, there was no significant difference between the groups in terms of how the drugs were applied, as single or repetitive doses (*p* = 0.254). Table 1 contains the details.

#### Reduction of Burst Suppression Ratio During Maintenance

The cumulative BSR (*p* = 0.828), the BSR duration (*p* = 0.687), and the maximum BSR (*p* = 0.926) during maintenance were not significantly different between groups (Figure 4B). We did not find a significant difference between the groups in the relative BSR either (*p* = 0.975). Table 1 contains the detailed parameter values.

#### Mean Arterial Blood Pressure Interventions and Mean Arterial Blood Pressure Values During Positive Burst Suppression Ratio Episodes

**FIGURE 5:**
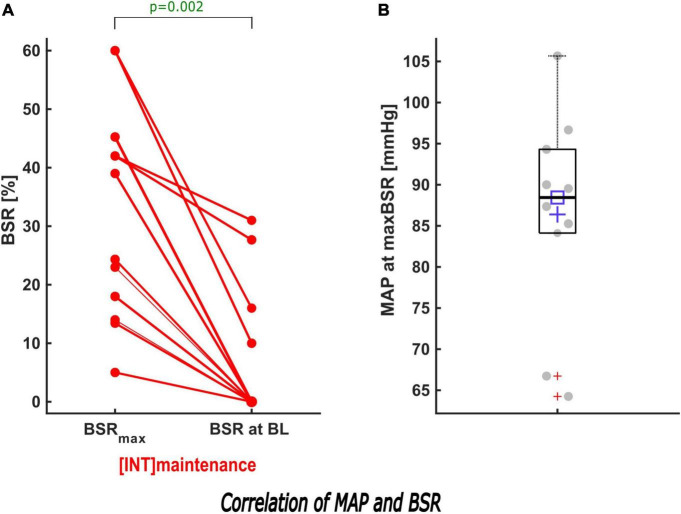
Description of the effect of MAP intervention on BSR during maintenance. **(A)** When the MAP was increased to its baseline value in INT during maintenance, a sub-analysis showed that the maximum BSR was reduced significantly (*p* = 0.002). In 7/11 patients, BSR was suppressed to 0. **(B)** The box and scatter plot shows the distribution of the MAP of all patients at their maximum BSR.

In the intervention group, we solely performed MAP intervention in 14/36 (39%) patients. In 19 (53%) patients we initially performed a MAP intervention and subsequently a MAC intervention (Supplementary Figure 1). When the MAP was elevated to its baseline value (*n* = 44), BSR > 0 was reduced and in 55% (24/44) fully eliminated. During induction, 17/33 MAP interventions resulted in BSR = 0, while the BSR decreased to 0 in 7/11 patients during maintenance (Figure 5 and Supplementary Table 3).

**FIGURE 6:**
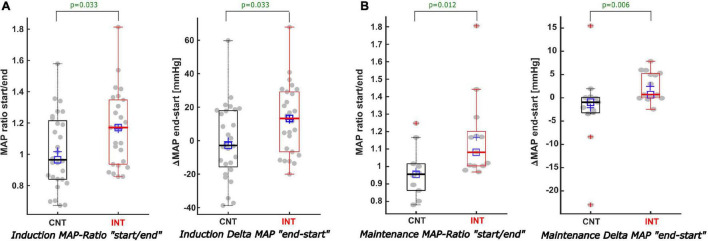
Description of the MAP values when BSR > 0 during induction and maintenance. **(A)** During induction **(A)** both the MAP ratio of “MAP at the start/end of the BSR” (A-left) and the Delta-MAP “end-start” (A-right) showed significant differences comparing CNT with INT. For INT the MAP was increased by 13 mmHg, while for CNT the MAP showed a decreasing trend of -2.9 mmHg [*p* = 0.033; AUC 0.33; CI (0.19; 0.48)]. **(B)** In contrast to the non-statistically significant trends of the cumulative BSR and duration of BSR during maintenance, the analysis of the MAP values during maintenance displayed–similar to the induction phase–considerable differences regarding both the MAP ratio (B-left) and the Delta-MAP (B-right) amongst both groups. MAP-ratio: CNT = 0.96 vs. INT = 1.08 (*p* = 0.012) and Delta MAP: CNT = -1 mmHg vs. INT = 0.7 mmHg [*p* = 0.006; AUC 0.19; CI (0.04; 0.38)].

**FIGURE 7:**
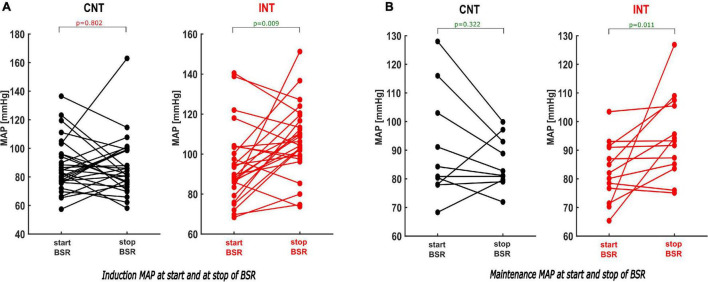
Paired analysis within the groups of the MAP values comparing the MAP at the start and the end of the BSR during induction and maintenance. **(A)** During induction the paired analysis within INT shows that the MAP by the end of BSR was elevated substantially by intervention [*p* = 0.009; Hedges’ *g* 0.68 (−1.25; −0.26)], while there was no significance within CNT [*p* = 0.802; Hedges’ *g* 0.03 (−0.38; 0.49)]. **(B)** Same findings were observed during maintenance: The intervention induced a considerable increase of the MAP at the end of BSR [*p* = 0.011; Hedges’ *g* −0.90 (−1.54; −0.42)]. In contrast with CNT, the MAPend often decreased compared to the MAPstart [*p* = 0.322; Hedges’ *g* 0.34 (−0.31; 0.89)].

During induction, MAPstart were not significantly different between the groups (*p* = 0.166). MAPend values were higher in the intervention group [CNT: 83 (75; 99) mmHg; INT:105 (99; 117) mmHg, *p* < 0.001, AUC = 0.19 (0.08; 0.32)]. The ΔMAP (MAPend-MAPstart) was –2.9 (–16; 18) mmHg in the control group and 13 (–7; 29) mmHg in the intervention group after MAP intervention [*p* = 0.033, AUC = 0.33 (0.19; 0.48)]. The relative MAP change from MAPstart to MAPend was also significantly stronger in the intervention group [CNT: 0.97 (0.84; 1.21); INT: 1.17 (0.94; 1.35), *p* = 0.033, AUC = 0.33 (0.19; 0.48)] (Figures 6A, 7A).

**FIGURE 8:**
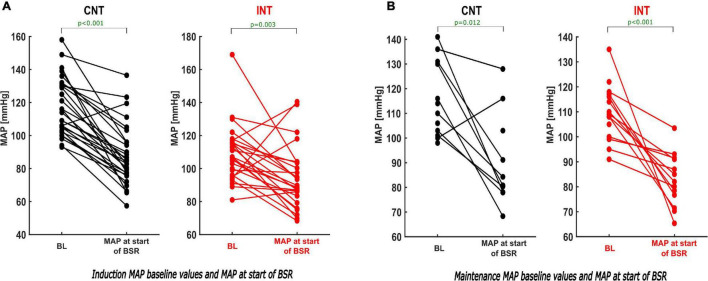
Paired analysis–comparison of the BL-MAP and MAP at the start of the BSR within the groups during induction and maintenance. During induction **(A)** and maintenance **(B)** the paired analysis of the MAP values within both groups (CNT–left; INT–right) demonstrates that the MAP at the start of the BSR was substantially lower than the individual baseline value. For absolute values see Table 1.

MAPstart was significantly lower than the baseline value in both groups [CNT: *p* < 0.001, Hedges’ *g* 1.64 (1.22; 2.30); INT: *p* = 0.003, Hedges’ *g* 0.81 (0.34; 1.48)] (Figure 8, Supplementary Table 4 and Table 1).

During maintenance the median MAP values at MAPstart and MAPend did not differ significantly between the groups. But MAP ratio “MAPstart/MAPend” [CNT: 0.96 (0.86; 1.02); INT: 1.08 (1.00; 1.20), *p* = 0.012, AUC = 0.19 (0.02; 0.39)] and ΔMAP [CNT: –0.96 (–3.2; 0.1) mmHg; INT: 0.7 (0.0; 5.2) mmHg, *p* = 0.006, AUC = 0.19 (0.04; 0.38)] did (Figures 6B, 7B). Similarly, the MAP ratio “MAPend/MAPBL” showed a statistical difference [CNT: 0.71 (0.67; 0.75); INT: 0.89 (0.75; 0.96), *p* < 0.001, AUC = 0.21 (0.04; 0.43)].

Incorporating the missing reductive effect of the BSR during maintenance, these findings of the MAP values may seem contradicting.

In the paired sub-analyses, we found that MAPstart was significantly lower than the baseline value within both groups [CNT: *p* = 0.012, Hedges’ *g* 1.55 (0.69; 3.71); INT: *p* < 0.001] (Figure 8B and Table 1).

#### Minimal Alveolar Concentration Values During Positive Burst Suppression Ratio Episodes During Maintenance

We did not observe a significant difference in ΔMAC or the MACstart to MACend ratio. Solely the paired analysis within groups showed a significant MAC reduction in the intervention group [MACstart: 1.05 (1; 1.25), MACend: 0.7 (0.6, 0.9); *p* = 0.047]. This result, like the MAP intervention, demonstrates a visible effect of a MAC intervention, however, without having an impact on the reduction of the total cumulative BSR during maintenance (Supplementary Figure 2 and Table 1).

#### Post-operative Delirium/Post-operative Neurocognitive Disorder

None of the patients was affected by POD during the first three post-operative days. A PACU-delirium was diagnosed in 28% of all patients regardless of positive or negative BSR (15/68 patients with positive BSR vs. 15/36 patients without a positive BSR). For patients with BSR > 0, 22% were diagnosed a PACU-delirium evenly distributed amongst the two groups [7/32 (CNT) vs. 8/36 (INT), *p* = 0.973].

Additionally, we compared the incidence of PACU-delirium, respectively, the intervention type, without finding a relevant difference between MAP and MAC interventions (“only MAP” 3/14 vs. “only MAC” 1/3; *p* = 0.659). Assuming that a BSR > 0 is more likely to cause delirium during maintenance, we also did not find significant differences between the two groups [3/14 (CNT) vs. 3/11 (INT); *p* = 0.734] (Supplementary Table 5 contains the details).

## Discussion

Over the past few decades of research, the occurrence of intraoperative BSupp has been predominantly described as a non-physiological, abnormal brain response to anaesthesia that may be related to post-operative complications. Some primarily observational trials described potential perioperative factors that can trigger BSupp; e.g., age, cerebral perfusion affected by the BP level, anaesthetic concentration, and increased cerebral sensitivity (Bruhn et al., 2000b; Sessler et al., 2012; Fritz et al., 2018). This suggests that altering the anaesthetic management reduces BSupp and consequently post-operative complications. There are still relatively few trials that analyse the potential of targeted anaesthetic interventions to reduce BSupp (Wildes et al., 2019).

About one third of all surgical procedures are performed in patients >65 years (Reiss et al., 1992; Etzioni et al., 2003). Numerous recent publications stated that predominantly elderly people (≥60 years) show intraoperative BSupp (Besch et al., 2011; Purdon et al., 2015). In this aged population, pNCD is one of the most common post-operative complications and has been related to serious acute and long-term consequences (Pompei et al., 1994; Ely et al., 2004; Salluh et al., 2015). The link between BSupp and pNCD remains unclear and is controversially discussed. Nevertheless, terms such as “common, costly, deadly” associated with pNCD illustrate the severe medical and economic relevance to probe its causes in reference to feasible anaesthetic courses of action. The ESA also underlines that POD is an expensive complication and multi-component interventions can reduce acute and long-term costs (Aldecoa et al., 2017). Their current guideline gives a grade A recommendation for monitoring anaesthetic depth to avoid excessive anaesthesia levels with BSupp. Our findings from a randomised interventional feasibility trial provide additional knowledge regarding strategies to reduce BSupp based on BP and anaesthetic concentration. Particularly the findings, respectively, the impact of haemodynamic interventions add to the hypothesis of underlying causes, such as hypoperfusion-induced BSupp and hence provide a magnificent incentive for larger, interventional clinical trials.

### Trigger Factors for Burst Suppression

In the past, few prospective randomised trials proposed that using processed EEG information during surgery may decrease the rate of post-operative delirium (Chan et al., 2013; Radtke et al., 2013). Wildes et al. (2019) published (one of) the first randomised interventional trials that investigated whether EEG-guided general anaesthesia in older adults undergoing either cardiac or non-cardiac surgery decreases POD incidence on post-operative days 1–5. While the POD rate was not reduced, although the BSR was successfully reduced by intervention, patients in the EEG-guided group received significantly less anaesthetics and spent significantly less time in EEG suppression (Wildes et al., 2019).

Sessler et al. (2012) showed that low values of the bispectral index (BIS), indicative of BSupp, are not simply related to the anaesthetic concentration, but also to low BP values. Moreover, their results showed that a “triple low” combination of low pEEG (BIS) parameters, low MAC and low MAP is associated with increased mortality. The pathomechanism of inadequate cerebral perfusion reflected by ischaemic suppression of brain metabolism is consistent with previous knowledge. Sessler et al. (2012) mention a potential connection between BSupp and hypotension, a frequent side effect of anaesthesia that seems independently associated with adverse perioperative outcomes in context with general anaesthesia. In general intraoperative hypotension has often been discussed with respect to multiple post-operative adverse outcomes (Wesselink et al., 2018). Several different definitions of intraoperative hypotension exist and there is no commonly accepted definition. Often a relative decrease–most frequently 20%–to the baseline of systolic arterial pressure or absolute values–primarily less than 80 mmHg–has been described (Bijker et al., 2007). Based on this controversy we decided to define an individual threshold based on the lowest BP recorded pre-operatively while the patient was in the hospital. This reflects the status of the individual patient and guarantees adequate blood flow in the brain for the individual patient conditioning sufficient cognitive function.

Our interventional protocol defined a targeted anaesthetic management with two separate interventions (MAP and MAC) to reduce BSupp. Overall we showed that targeted anaesthetic management based on EEG-monitoring significantly reduces the total cumulative BSR and the total BSR duration. Our intervention effectively reduced the BSR, regardless of demographic influencing factors or anaesthetic doses. In contrast to Wildes et al. (2019), the two-armed interventional approach allowed a more detailed analysis of the association between BP and BSR with interesting findings. As expected, the mean MAP was significantly higher after a MAP intervention compared to the control group. More remarkable, however, is the fact that a MAP intervention reduced the maximum BSR in all patients. In 55% of all MAP interventions, we could reduce the BSR to 0% without an additional MAC intervention. During a continuous rate of anaesthetics during maintenance, MAP interventions eliminated BSupp in 7/11 patients (64%). The very small sample size of the cases “only MAP intervention” does not allow a statistical plausible sub-analysis yet. But for now, we aimed to underline the importance of investigating the mechanistic role of hypotension and hence cautiously conclude that treating intraoperative hypotension considerably reduces the BSR. Although this does not prove a causality between intraoperative hypotension, cerebral hypoperfusion, and the occurrence of BSupp, it shows a substantial impact. Eventually these findings provide an incentive for larger, interventional trials—primarily focusing on BSupp during maintenance—which are needed before recommendation for intervention strategies can be made.

The hypothesis to consider hypotension-induced cerebral hypoperfusion as possible pathophysiological cause for BSupp seems also of interest in the context of previous and current debate about neurophysiological-metabolic models of BSupp. These models imply that down-regulated neuronal spiking activity coupled with decreased cerebral blood flow or metabolic rates to stabilise properties of ATP-gated potassium channels may lead to a distinctive suppression EEG (Ching et al., 2012).

### Burst Suppression and Post-operative Neurocognitive Disorders

The current discussion about a potential coherence of BSupp and pNCD remains controversial. Apart from a few recent interventional trials (ENGAGES) that did not find a correlation between intraoperative BSupp and POD, the majority of earlier publications pointed towards decreased incidences of POD in patients without (processed) BSupp (Radtke et al., 2013; Soehle et al., 2015; Fritz et al., 2016; Hesse et al., 2019). In our trial, 28% out of all patients developed a PACU delirium irrespective of whether they showed BSR > 0 or not. None of the patients suffered from POD. With a focus on the patients with BSR > 0 (*n* = 68), we diagnosed 22% with PACU delirium evenly distributed amongst the two groups, without showing a relevant difference. Neither did we find any significant differences within the intervention group concerning the intervention type. Although BP management considerably affected the (maximum) BSR, patients who benefitted from the haemodynamic intervention did not suffer less from delirium as did patients who only received reduced anaesthetic doses. These findings generally resemble the latest results of the ENGAGES trial. At this stage it is necessary to consider that our statistical analyses simply focused on the trend data of the processed EEG, hence, these findings may not be fully interpreted. Additionally, the overall low incidence or a rather small study population make further interpretation unreasonable, particularly, because we did not primarily focus our investigation on POD. Finally, we have often discussed the remaining ambiguity whether the results of the neurocognitive assessment may be more indicative of a delayed (neuro-cognitive) emergence/recovery rather than a definite diagnosis of delirium.

### Strengths and Limitations

Strengths of our investigations were (i) the two-armed interventional approach with two pragmatic adjusting parameters to reduce intraoperative BSR, (ii) the focus on elderly patients (>60) without pre-existing neuropsychiatric conditions for a homogenous patient cohort and reduced potential biasing factors, and (iii) the clear definition of the MAP intervention based on the individual patient’s lowest MAP.

One limitation of our study is the analysis of pEEG data, i.e., the BSR. This may be considered a major limitation regarding a more conclusive interpretation, especially since the EEG-based monitoring systems may underestimate the occurrence of BSupp and there may be contradicting information between BSR and the “depth of anaesthesia” index (Muhlhofer et al., 2017; Georgii et al., 2020). Undetected BSupp may lead to (very) high SE values (Hart et al., 2009). A quick analysis of the data revealed that 57% (59/104) of the patients had SE ≥ 80 as the maximum SE during maintenance. The median value of maximum SE in all patients was 85 (72, 99) (Supplementary Figure 2). These findings highlight possible limitations of pEEG monitoring, especially, since the BSupp EEG is affected by factors such as age and substance (Fleischmann et al., 2018; Kratzer et al., 2020).

Further, the propofol bolus for induction resulted in a higher BSR incidence than during maintenance with continuous application of the anaesthetic. The rapid aggregation of various influences makes it difficult to differentiate the effects during induction: a highly probable anaesthetic-induced hypotension requiring an intervention coincides with rapid pharmacodynamic changes of anaesthetics, e.g., changing to inhaled anaesthetics while intravenous anaesthetics are wearing off. The use of target-controlled infusion systems may have enabled a more consistent comparison of BSupp findings during induction and advanced analyses of pathological aspects such as the frailty of each individual’s brain. Interestingly, Hesse et al. (2019) recently discussed that visually detected BSupp during induction is less associated with post-operative delirium compared to BSupp during maintenance.

Finally, this trial examined a relatively small sample size which is why the analysis of the data especially with respect to the evaluation of the interventions appears limited. Supplementary Table 3 illustrates three distinct categories of interventions being considered, majorly conducted during induction. For a more detailed sub-analysis a larger sample size is required. Hence the results incentivise to conduct lager, interventional clinical trials in the future.

## Conclusion

Based on our hypotheses of the aetiology of intraoperative BSupp this study focused on MAP and MAC interventions as these strategies can easily be performed on the one hand and adjusted with well-established clinical methods on the other hand. Our results allow us to conclude that targeted interventions can reduce the total cumulative amount, the duration, and the maximum rate of BSR. More relevant, we showed that a solitary MAP intervention reduced the BSR, coinciding with current reflections about hypotension-induced cerebral hypoperfusion. However, to fully understand the pathophysiological background, to reason a potential causality between cerebral hypoperfusion and BSupp and hence to prove the potential effectiveness of MAP intervention when BSR > 0, further investigations with larger study populations are required.

## Data Availability Statement

The original contributions presented in the study are included in the article/Supplementary Material, further inquiries can be directed to the corresponding author.

## Ethics Statement

The study involving human participants was reviewed and approved by Local Ethics Committee of the Technical University of Munich, Department of Medicine (Chairperson Georg Schmidt). The patients/participants provided their written informed consent to participate in this study.

## Author Contributions

M-TG was mainly responsible for the implementation, completion, and data evaluation of this study project. MK helped with the technical setup as well as the statistical evaluation. AF and JS supported the recruitment process and data collection. GS and SP were responsible for the whole project and trial. All authors contributed to the article and approved the submitted version.

## Conflict of Interest

The authors declare that the research was conducted in the absence of any commercial or financial relationships that could be construed as a potential conflict of interest.

## Publisher’s Note

All claims expressed in this article are solely those of the authors and do not necessarily represent those of their affiliated organizations, or those of the publisher, the editors and the reviewers. Any product that may be evaluated in this article, or claim that may be made by its manufacturer, is not guaranteed or endorsed by the publisher.

## Footnotes

1
https://www.socscistatistics.com
