# Supplementary material for: Targeted Interventions to Increase Blood Pressure and Decrease Anaesthetic Concentrations Reduce Intraoperative Burst Suppression: A Randomised, Interventional Clinical Trial
Source: Front Syst Neurosci. 2022 Mar 4;16:786816. doi: 10.3389/fnsys.2022.786816 (PMC8931826; doi:10.3389/fnsys.2022.786816)
Supplement: Supplementary file 1 [file Data_Sheet_1.pdf]

Supplementary Material

All Patients with positive BSR n=68			
Preoperative patient characteristics			
	CNT	INT	p-Value
Age	73 [67; 79]	73 [68; 79]	0.768
Sex			
female	21 (30%)	23 (30%)	0.983
male	12 (17%)	13 (19%)	
BMI	27 [25; 28]	26 [25; 29]	0.957
ASA status (1,2,3,4)			
1	3 (4%)	1 (1%)	0.527
2	16 (23%)	18 (26%)	
3 (+4)	14 (20%)	17 (24%)	
MAP baseline value	113 [103; 130]	108.5 [100; 116]	0.156

Table S1 - Description of the preoperative patient characteristics

Surgical disciplines	Quantity
Orthopaedics	45
Urology	34
Visceral surgery	14
Traumatology	7
Neurosurgery	3
Sports orthopaedics	1
Gynaecology	1
Vascular surgery	1

Table S2 - Distribution of surgical disciplines

Distribution of blood pressure adjustments vs anaesthetic reduction amongst all 36 patients in the INT-group			
	Only MAP intervention	Only anaesthetic-intervention	Both
Number of patients	14 (39%)	3 (8%)	19 (53%)
Absolute quantity of MAP interventions and the effect on BSR			
	Total number of MAP interventions	Reduction of BSR after MAP intervention	Elimination of BSR after MAP intervention
Quantity of all MAP interventions	44	44 (100%)	24 (55%)
Quantity of MAP interventions during maintenance	11	11 (100%)	7 (64%)

Table S3 – Quantity of MAP interventions and their effect on BSR

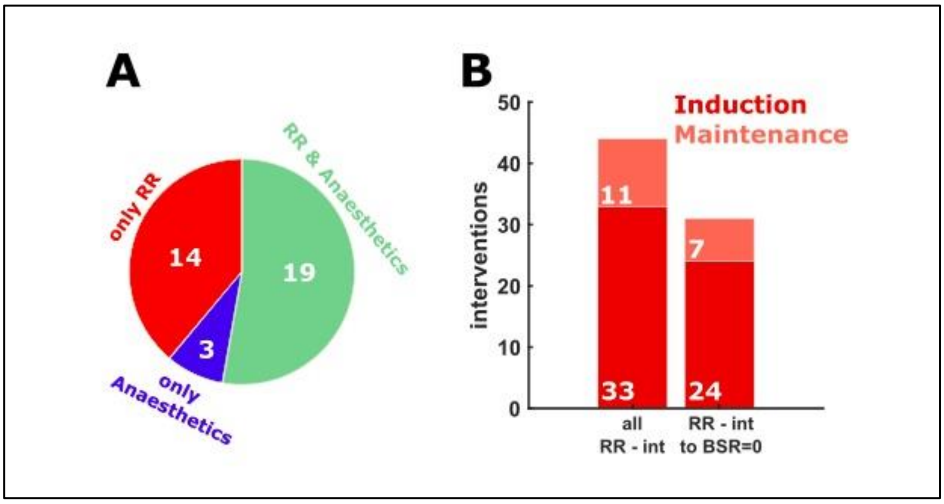

Figure S1 - *(A) Total number of patients in the intervention group receiving either MAP, anaesthetic, or both interventions. (B) Quantity of MAP interventions and their effect on BSR.*

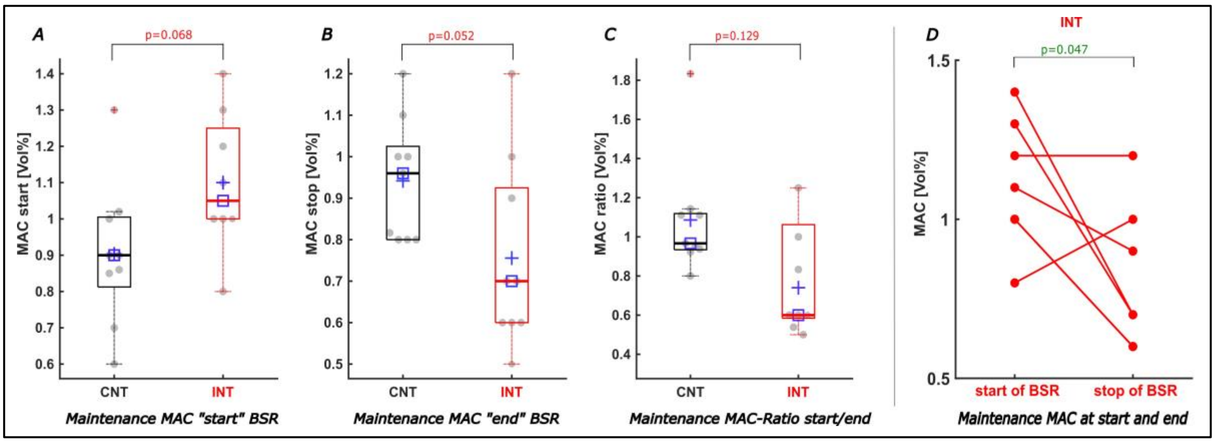

Figure S2 - Description of the MAC values at positive BSR during maintenance
With respect to the anaesthetic doses and effects of the intervention the plots show the comparison of the ***MAC*** *at the* ***start*** *of the BSR* ***(A)***, ***MAC*** *at the* ***end*** *of the BSR* ***(B)*** *and the* ***MAC ratio*** *"start/end"* ***(C)*** *between the groups during maintenance. The MAC concentration corresponds to the end-tidal volume concentration in [Vol%]. None of these comparisons showed relevant differences. Simply the paired analysis within the intervention group (MAC start to end)* ***(D)*** *demonstrates an effective reduction of the MAC.*

Paired statistical analysis within the groups		
**Induction**		
	CNT	INT
MAP start to end of BSR		
p-Value[a]	0.802	0.009
Hedges' g	0.03 [-0.38; 0.49]	-0.68 [-1.25; -0.26]
MAP BL to start of BSR		
p-Value[a]	<0.001	0.003
Hedges' g	1.64 [1.22; 2.30]	0.81 [0.34; 1.48]
MAP BL to end of BSR		
p-Value[a]	<0.001	0.675
Hedges' g	1.56 [0.97; 2.50]	0.12 [-0.39; 0.61]
**Maintenance**		
MAP start to end of BSR		
p-Value[a]	0.322	0.011
Hedges' g	0.34 [-0.31; 0.89]	-0.90 [-1.54; -0.42]
MAP BL to start of BSR		
p-Value[a]	0.012	<0.001
Hedges' g	1.55 [0.69; 3.71]	2.31 [1.69; 3.64]
MAP BL to end of BSR		
p-Value[a]	<0.001	0.013
Hedges' g	1.56 [0.97; 2.50]	0.12 [-0.39; 0.61]
MAC start to end of BSR		
p-Value[a]	0.742	0.047
Hedges' g	-0.20 [-1.09; 0.48]	1.42 [0.52; 3.42]

Table S4 - ***Paired statistical analysis within the groups****; [a]Wilcoxon signed-rank test*

	PACU delirium	No PACU delirium	p-Value
Total number of patients (n=104)			
INT (n=50)	15 (14%)	35 (34%)	0.643
CNT (n=54)	14 (14%)	40 (38%)	
Positive BSR (n=68)			
INT (n=36)	8	28	0.973
CNT (n=32)	7	25	
BSR during maintenance			
INT (n=11)	3	8	0.734
CNT (n=14)	3	11	
No BSR			
INT (n=14)	7	7	0.419
CNT (n=22)	8	14	
INT – Incidence of delirium in context with type of intervention (MAP vs anaesthetics)			
Only MAP intervention (14)	3	11	0.659
Only anaesthetic reduction (3)	1	2	

Table S5 - Incidence of PACU delirium
PACU delirium among all patients included in the trial, only patients with positive BSR (during maintenance) or without positive BSR and in context with the type of intervention (MAP vs anaesthetics) applied.
